# TLR3 Ligand PolyI:C Prevents Acute Pancreatitis Through the Interferon-β/Interferon-α/β Receptor Signaling Pathway in a Caerulein-Induced Pancreatitis Mouse Model

**DOI:** 10.3389/fimmu.2019.00980

**Published:** 2019-05-03

**Authors:** Chaohao Huang, Shengchuan Chen, Tan Zhang, Dapei Li, Zhonglin Huang, Jian Huang, Yanghua Qin, Bicheng Chen, Genhong Cheng, Feng Ma, Mengtao Zhou

**Affiliations:** ^1^Key Laboratory of Diagnosis and Treatment of Severe Hepato-Pancreatic Diseases of Zhejiang Province, Department of Surgery, First Affiliated Hospital of Wenzhou Medical University, Wenzhou, China; ^2^Suzhou Institute of Systems Medicine, Peking Union Medical College, Chinese Academy of Medical Sciences, Suzhou, China; ^3^Center for Systems Medicine, Institute of Basic Medical Sciences, Peking Union Medical College, Chinese Academy of Medical Sciences, Beijing, China; ^4^Department of Emergency, First Affiliated Hospital of Soochow University, Suzhou, China; ^5^Department of Laboratory Diagnosis, Changhai Hospital, The Second Military Medical University, Shanghai, China

**Keywords:** acute pancreatitis, TLR3 ligands, polyI:C, reactive oxygen species, type I interferon, IFN-β, neutrophil infiltration

## Abstract

Acute pancreatitis (AP) is a common and devastating inflammatory disorder of the pancreas. However, there are still no effective treatments available for the disease. Therefore, it is important to discover new therapeutic targets and strategies for better treatment and prognosis of AP patients. Toll-like receptor 3 (TLR3) ligand polyI:C is a double-stranded RNA mimic that can be used as an immune stimulant. Our current study indicates that polyI:C exerted excellent anti-inflammatory effects in a caerulein-induced AP mouse model and taurocholate-induced pancreatic acinar cell line injury model. We found that polyI:C triggers type I interferon (IFN) production and downstream IFN-α/β receptor (IFNAR)-dependent signaling, which play key roles in protecting the pancreas from inflammatory injury. Knockout of IFN-β and IFNAR in mice abolished the preventive effects of polyI:C on caerulein-induced AP symptoms, which include pancreatic edema, neutrophil infiltration, the accumulation of reactive oxygen species (ROS), and inflammatory gene expression. Treating pancreatic acinar 266-6 cells with an IFNAR inhibitor, which blocks the interaction between type I IFN and IFNAR, diminishes the downregulation of oxidative stress by polyI:C. Additionally, a subsequent transcriptome analysis on the role of polyI:C in treating pancreatitis suggested that chemotaxis of neutrophils and the production of ROS were inhibited by polyI:C in the pancreases damaged by caerulein injection. Thus, polyI:C may act as a type I IFN inducer to alleviate AP, and it has the potential to be a promising therapeutic agent used at the early stages of AP.

## Highlights

- PolyI:C significantly alleviates caerulein-induced acute pancreatitis;
- PolyI:C attenuates acute pancreatitis-related oxidative stress;
- PolyI:C protects mice from pancreatic injuries in an IFN-β-dependent manner;
- TLR3 agonists would be promising therapeutic agents for acute pancreatitis.

## Introduction

Acute pancreatitis (AP) is an inflammatory condition of the pancreas, that frequently leads to systemic inflammatory response syndrome (SIRS), multiple organ dysfunction syndromes (MODS), and even death without early intervention (1). The mortality of individuals with AP-MODS exceeds 20%, and the quality of life of those who experience the devastating inflammatory disease is significantly worse than that of the general population (2). Research from many groups including our own have shown that pancreatitis involves pancreas edema, inflammatory cell infiltration, and high levels of serum amylase and lipase (3, 4). Meanwhile, reactive oxygen species (ROS) generated by injured pancreatic acinar cells and infiltrated immune cells are key factors in the progression of pancreatitis (5). Therefore, novel therapeutic strategies or pharmaceutical interventions to decrease the accumulation of ROS, limit local inflammatory damage, and accelerate the recovery of the injured pancreas are needed urgently.

Previous studies on AP have suggested that damage of pancreatic acinar cells results in the upregulation of inflammatory cytokines and chemokines (6). They initiate the inflammatory response and the recruitment of inflammatory cells, which leads to pancreatitis (7). Activated inflammatory cells contribute to subsequent pancreatic injury through the generation of ROS (8). As the ROS accumulate, so does the demand for the anti-superoxide response (9). Heme Oxygenase-1 (HO-1), a well-known anti-oxidant molecule, is always highly expressed during the inflammatory response (10). High levels of HO-1 are an indicator of severe tissue damage and oxidative stress, which require a stronger anti-oxidant reaction (10). Thus, HO-1 could be used as an indicator for measuring the severity of damage and inflammation in AP.

PolyI:C is synthetic double-stranded RNA, which is used as a viral RNA mimic to induce type I IFN and trigger antiviral immunity-based pathways in the host. PolyI:C is considered to be a pathogen-associated molecular pattern (PAMP) because it interacts with Toll-like receptor 3 (TLR3) and activates TLR3-dependent downstream signaling (11, 12). Pattern recognition receptors (PRRs) such as TLR9 and NLRP3 are required for the development of inflammation in AP, and their antagonism could provide a new therapeutic strategy for treating AP (13). Moreover, TLR3 ligand polyI:C treatment significantly decreases the mortality and liver injury caused by injection of lipopolysaccharide (LPS) in the presence of D-galactosamine (D-GalN) in C57BL/6 mice (14), which has driven us to test the anti-inflammatory role of polyI:C during AP progression. As a downstream product of the TLR3-TRIF-TBK1-IRF3 signaling axis, type I IFN has been demonstrated as an immune mediator that also exerts an anti-inflammatory function as well as its antiviral activity (15). IFN-α and IFN-β are key members of the type I IFN family in combating virus infection and regulating immune function, and IFN-β is the initial response of type I IFN produced at the early stages of infection or following PAMP stimulation (16, 17). Subsequently, more IFN-β and IFN-α is produced at the later stages of infection via a positive feedback loop (16–18). It was demonstrated that IFN-β counteracts the overexpressed ICAM-1 in cultured brain-derived microvascular endothelial cells (BMEC) incubated with TNF-α, which leads to a reduction in the adhesion of leukocytes to blood vessels and thus a reduced inflammatory reaction (19). Many other similar observations support the conclusion that IFN-β participates in attenuating the acute inflammatory response because of its regulatory effect (20, 21). It is interesting to investigate whether polyI:C has the potential to limit detrimental and pathological immune responses which lead to tissue damage in a caerulein-induced pancreatitis mouse model, via the IFN-β/IFNAR signaling pathway.

In this study, we have found that polyI:C pretreatment prevents caerulein-induced pancreas edema, neutrophil infiltration, the accumulation of ROS, and inflammatory gene expression in the AP mice models. PolyI:C-triggered IFN-β production and downstream IFNAR signaling activation are required for the suppressive effect of polyI:C in the caerulein-induced AP model. Our study has not only demonstrated the protective role of polyI:C in limiting AP, but has also suggested a potential application of TLR3 ligands in the treatment of AP.

## Materials and Methods

### Mice and Reagents

*Ifnb*^−/−^ mice and *Ifnar1*^−/−^ mice were gifted from Genhong Cheng Laboratory (University of California, CA, USA). *Tlr4*^−/−^ mice were purchased from Model Animal Research Center (Nanjing, China). Wild-type (WT) C57BL/6 mice were acquired from Vital River Laboratory Animal Technology (Beijing, China). All the mice were maintained in the specific pathogen-free (SPF) environment at Suzhou Institute of Systems Medicine (ISM) under a controlled temperature (25°C) and a 12-h day/night cycle. Male 8–10-week-old mice were used in all the experimental AP mice models. All mice experiments were undertaken in accordance with the US National Institutes of Health Guide for the Care and Use of Laboratory Animals, with the approval of the Scientific Investigation Board of ISM, Suzhou. Antibodies against HO-1 (#70081) and GAPDH (#5174) were from Cell Signaling Technology (Danvers, MA). NQO1 antibody (#ab2346) and KEAP1 antibody (#ab119403) were from Abcam (Cambridge, MA). Caerulein and IFNAR inhibitor were from MCE (Monmouth Junction, NJ). L-Arginine was from Sigma-Aldrich (St. Louis, MO). PolyI:C was from Thermo Fisher Scientific (Waltham, MA). Recombinant mouse IFN-β was from R&D Systems (Minneapolis, MN). Taurocholate was from SolarBio (Beijing, China).

### Experimental AP Mice Models

Before the induction of experimental AP, the mice fasted for 8 h and were intraperitoneally injected with 100 μl polyI:C (10 mg/kg) or saline as a control. After 1 or 8 h of polyI:C/saline injection, the mice were intraperitoneally administered 200 μl caerulein (200 μg/kg) 10 times or L-Arginine (2.5 g/kg) twice, 1 h apart. Mice were sacrificed at 24 h post the initial induction of the AP, and the serum and pancreas were collected for further analysis. The pancreases were placed in 4% paraformaldehyde for histological analysis or stored in RNAlater (QIAGEN, Düsseldorf, Germany) for RNA extraction. In addition, fresh pancreases were isolated for analyzing inflammatory immune cell infiltration via flow cytometry or snap-frozen for protein extraction for western blotting analysis.

### Cell Culture and Flow Cytometry Analysis

The mouse pancreatic acinar 266-6 cell line was purchased from ATCC (Manassas, VA) and cultured in RPMI-1640 medium supplemented with 10% FBS (Gibco, ThermoFisher Scientific) 100 IU/ml penicillin and 100 μg/ml streptomycin under the conditions of 37°C and 5% CO_2_. To induce pancreatitis *in vitro*, the 266-6 cells were stimulated with 0.5 mM taurocholate in the presence of polyI:C (1 μg/ml) or IFN-β (200 U/ml). Twenty-four hours later, cells were collected for the following Western blot assay, DCFH-DA staining, and flow cytometry analysis. To analyze the infiltrated innate immune cells during caerulein-induced AP, pancreases of mice were immediately harvested and incubated with 1 mg/ml collagenase D (ThermoFisher Scientific, Waltham, MA), and minced into small pieces on ice. Single pancreatic cell suspensions were first stained with Fc blocking antibody, then immune-labeled with fluorochrome-conjugated antibodies in PBS supplemented with 2% heat-inactivated FBS (Gibco, Thermo Fisher Scientific); isotype controls were also included. Antibodies Alexa Fluor 647-conjugated anti-CD11b, PE-conjugated anti-F4/80, and PerCP/Cy5.5-conjugated anti-Ly6G were purchased from BioLegend (San Diego, CA). Flow cytometry analysis was performed on a Life Launch Attune NxT Flow Cytometer (ThermoFisher Scientific, Waltham, MA) after gating the living cells. Data were analyzed using FlowJo software (version 10.0).

### Pancreas Histological Examination and Neutrophil Immunohistochemistry

Pancreatic tissues were collected 24 h after the induction of AP by caerulein and were fixed in 4% paraformaldehyde in PBS. Paraffin embedded tissues from each mouse were sectioned at 5 μm and these were followed by H&E staining. Immunohistochemistry for neutrophil marker MPO (#ab9535, Abcam, San Francisco, CA) was performed on saline or caerulein-induced AP tissues. Briefly, 5 μm thick paraffin sections of formalin-fixed paraffin-embedded pancreatic tissue were fixed in dimethylbenzene, quenched with 3% H_2_O_2_, and blocked with goat serum. After three washes with PBS, the sections were treated with anti-MPO primary antibody (1:50) overnight. Then, the sections were incubated with horseradish-peroxidase–conjugated secondary antibody for 1 h. Finally, color was developed using DAB as peroxidase substrate, and the slides were counterstained with hematoxylin for bright field microscopy. The degree of pancreatic injury was evaluated by light microscopy in 200X magnification over five separate fields. The severity of pancreatitis was scored mainly based on the description in the previous study (22), and is listed in Supplementary Table 2.

### Enzymatic Method Measurement of Serum Amylase and Lipase

Blood from experimental AP mouse models was centrifuged at 4,000 rpm, 1,500 g for 10 min at 4°C to separate the serum. Serum samples were diluted to the appropriate concentration and incubated with corresponding reagents in kit, then amylase and lipase activities were measured by commercial α-Amylase Assay Kit (C016-1) and Lipase Activity Kit (A054-2), respectively, according to the manufacturer's instructions (Nanjing Jiancheng Bioengineering Institute, Nanjing, China).

### Intracellular ROS Detection

Intracellular ROS intensity was measured by Reactive Oxygen Species Assay Kit (Beyotime, Shanghai) as described in the manufacturer's instructions. DCFH-DA fluorescence was measured by using Life Launch Attune NxT Flow Cytometer (Thermo Fisher Scientific, Waltham, MA, USA). Images were taken using Nikon Eclipse TI fluorescence microscope (Nikon Corporation, Tokyo). Data were analyzed using FlowJo software (version 10).

### Protein Extraction and Western Blotting

Snap-frozen pancreatic tissues were homogenized and resuspended in the buffer containing 4% sodium dodecyl sulfate (SDS) and 100 mM Tris-HCl. For immunoblot analysis, pancreatic tissue and 266-6 cells were collected in Triton lysis buffer (50 mM Tris-Cl, pH 7.5, 150 mM NaCl, 1 mM EDTA, 1% Triton X-100, and 5% glycerol) containing complete protease inhibitors (Roche). Protein concentrations of the extracts were measured with a BCA assay (ThermoFisher Scientific) and equalized with the lysis buffer. Equal amounts of the extracts were loaded and subjected to SDS-PAGE, transferred onto PVDF membranes (Millipore), and then blotted with enhanced chemiluminescence (Pierce) or Odyssey Imaging Systems (LI-COR Biosciences).

### RNA Isolation and Quantitative PCR (qPCR)

RNA was isolated from pancreatic tissue which was stored in RNAlater or 266-6 cells using TRIzol (ThermoFisher Scientific) according to the manufacturer's instructions. Following RNA concentration, quantitation of 1 μg of RNA was used to make cDNA using PrimeScript™ RT Reagent Kit for RT-PCR (Takara Shuzo Co., Tokyo) according to the manufacturer's instructions. Real time PCR analysis was performed using cDNA in the Roche 480 instrument using SYBR from Toneker Biotech (Suzhou, Jiangsu). The relative mRNA expression level of genes was normalized to the internal control ribosomal protein gene *Rpl32* by using 2^−ΔΔCt^ cycle threshold method (23). The primer sequences for qPCR were from the primer bank (24), and sequences are listed in Supplementary Table 1.

### RNA-Sequencing Data Acquisition, Quality Control, and Processing

Total pancreas RNA was extracted from caerulein-induced experimental AP mice models. RNA concentration was quantified using a Qubit 2.0 Fluorometer (Thermo Fisher). The quality of extracted RNA was evaluated using an Agilent Technology 2100 Bioanalyzer. RNA libraries were constructed using a TruSeq Stranded mRNA Sample Prep Kit (Illumina) according to the manufacturer's guidelines. The quantity and quality of the libraries were also assessed by Qubit and Agilent 2100 Bioanalyzer, respectively; their molar concentration was validated by qPCR for library pooling. Libraries were sequenced on the HiSeq X10 using the paired end 2^*^150 bp, dual-index format. For RNA-Seq data analysis, Trimmomatic was used to remove Illumina sequencing adapters within raw reads of every sample, trim low quality bases of both read ends (with parameters LEADING:3 TRAILING:3 SLIDINGWINDOW:4:15) and drop one read if its length is <36 bp. Secondly, the clean reads were mapped to mouse mm 10 reference genome with STAR, and the alignment bam files were used as htseq-count (command of python package HTSeq) input to get read counts of genes. Finally, DESeq2 was used to identify DEGs (*p* ≤ 0.05, FC ≥ 2) based on raw read counts. For DEGs, Ingenuity Pathway Analysis (IPA) and GO biological process were performed by Fisher's exact test, the enrichment *p*-values of which were corrected by the Bonferroni method. RNA-Seq data have been deposited to Gene Expression Omnibus (GSE119844).

### Statistical Analysis

The data represent the mean of at least three independent experiments, and error bars represent standard error or standard deviation of the mean. Statistical analysis was performed by unpaired 2-tailed student *t* test or one-way analysis of variance (ANOVA) followed by Tukey's multiple-comparison tests using GraphPad Prism (version 5; GraphPad Sofware Inc.). *p* < 0.05 was considered as a statistically significant difference.

## Results

### PolyI:C Prevents AP in WT Mice

**Figure 1:**
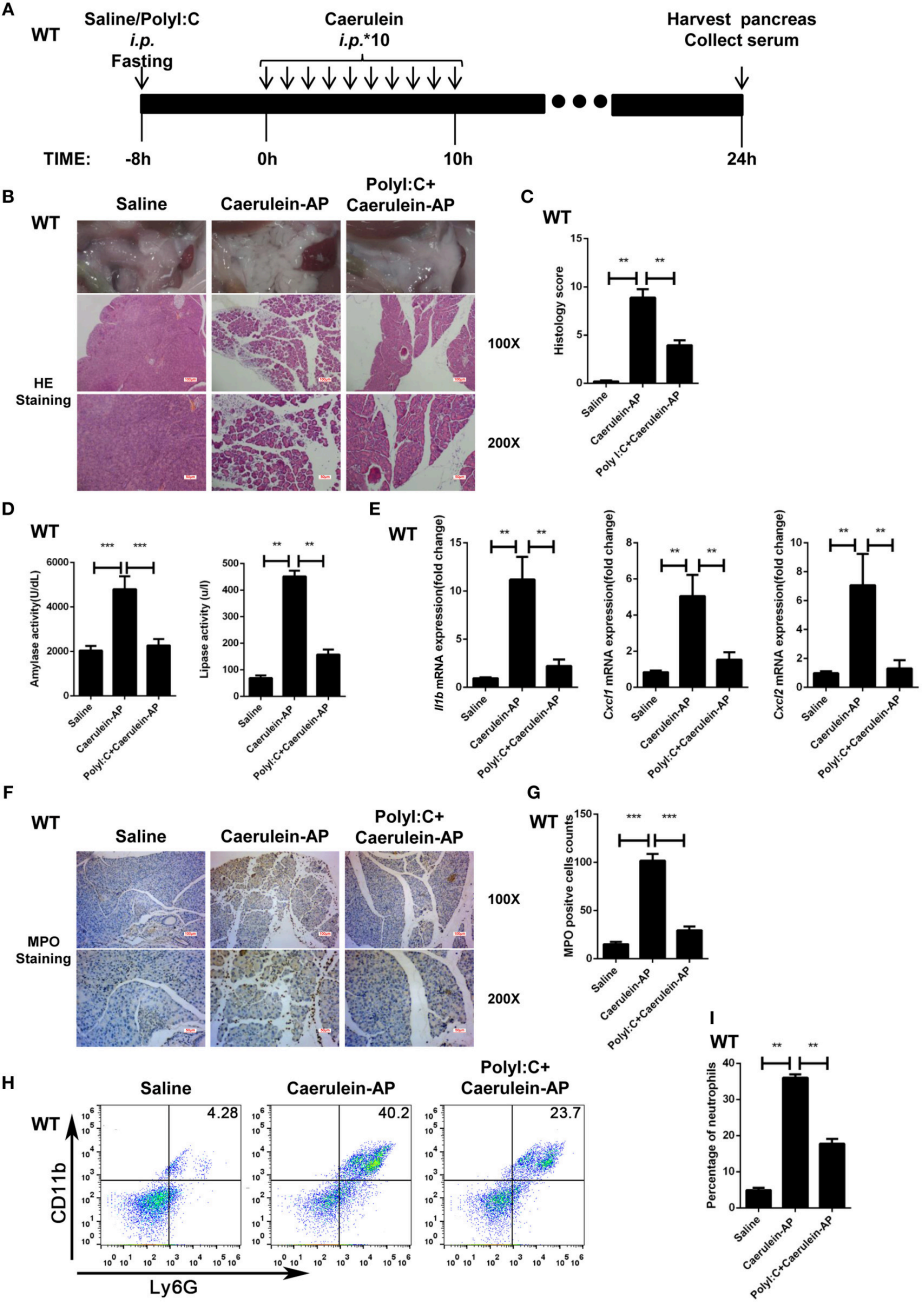
PolyI:C prevents caerulein-induced AP in the WT mice. **(A)** Schematic diagram of the caerulein-induced experimental AP mouse model. Saline or polyI:C (10 mg/kg) was intraperitoneally administrated 8 h prior to the induction of AP. **(B)** Histopathological examination of the effect of polyI:C on WT caerulein-induced experimental AP mouse models. Top panel: gross observation; middle panel: H&E staining, 100X magnification; bottom panel: H&E staining, 200X magnification. **(C)** Histology scores of pancreatitis were evaluated and compared after observing five separate fields. **(D)** Activities of the serum amylase (left) and lipase (right) from Saline, Caerulein-AP, and PolyI:C+Caerulein-AP WT mice were compared via enzymatic methods. **(E)** mRNA expression levels of *Il1b, Cxcl1*, and *Cxcl2* genes in the pancreatic tissue from Saline, Caerulein-AP, and PolyI:C+Caerulein-AP WT mice were detected by RT-qPCR and normalized to *Rpl32*. **(F)** Neutrophil infiltrations in the pancreases from WT caerulein-induced experimental AP mouse models were measured and compared by MPO staining. Top panel: 100X magnification; bottom panel: 200X magnification. **(G)** MPO^+^ cells were counted and compared after observing five separate fields. **(H)** Pancreases neutrophils from the Saline, Caerulein-AP, and PolyI:C+Caerulein-AP WT mice were analyzed by flow cytometry. CD11b^+^Ly6G^+^ cells were considered as neutrophils. **(I)** The percentage of neutrophils from indicated groups were calculated and compared. Data of **(B,F,H)** are representative of three independent experiments. Data of **(C,G)** are shown as mean ± SD (*n* = 5) from one representative experiment. Data of **(D,E,I)** are shown as mean ± SEM from at least three independent experiments. ^*^p < 0.05, ^**^p < 0.01, ^***^p < 0.001, one-way ANOVA test.

The protective effect of polyI:C on pancreatitis was evaluated in the caerulein-AP experimental mouse model at 24 h after the first injection of caerulein (Figure 1A). Administration with caerulein led to dramatic pathological changes including pancreas edema, inflammatory cell infiltration, and tissue necrosis. However, polyI:C pretreatment prevents these pancreatitis symptoms induced by caerulein injection (Figures 1B,C). Consistently, polyI:C pretreatment inhibited the elevation of serum amylase and lipase in the caerulein-AP mouse model (Figure 1D). PolyI:C also suppressed the induction of inflammatory cytokine and chemokine gene expression including IL-1β, CXCL1, and CXCL2 in the injured pancreases from caerulein-AP mice (Figure 1E). Neutrophils were recruited into the injured pancreases during pancreatitis progression, whereas notably fewer MPO^+^ cells were observed in the injured pancreases from caerulein-AP mice pretreated with polyI:C. This suggests polyI:C pretreatment inhibited neutrophil infiltration, a key cause of AP (Figures 1F,G). These immunohistochemistry results were verified by flow cytometry assay. Significantly fewer infiltrated CD11b^+^Ly6G^+^ cells (neutrophils) were detected in the pancreases from polyI:C-pretreated AP models (Figures 1H,I), while the infiltrated CD11b^+^F4/80^+^ cells (macrophages) were not affected (Supplementary Figure 1). These data indicate that polyI:C pretreatment specifically inhibits neutrophil infiltration rather than other immune cells such as macrophages.

Additionally, we checked whether polyI:C pretreatment affects pancreas homeostasis in a non-pathogenic saline-injected mouse model instead of in the AP model (Supplementary Figure 2A). No significant differences to pancreas edema, serum amylase and lipase levels, inflammatory cytokine and chemokine gene expression or neutrophil and macrophage infiltration were observed between the saline-pretreated and polyI:C-pretreated groups (Supplementary Figures 2B–G). These results suggest that polyI:C administration to pancreases is potentially safe.

We also tested the protective effect of polyI:C in L-arginine-induced AP mouse models (Supplementary Figure 3A). PolyI:C pretreatment significantly reduced the pancreatic injury caused by L-Arginine injection (Supplementary Figure 3B). Consistently, induction of serum amylase and lipase by L-Arginine was also attenuated in the polyI:C-pretreated group (Supplementary Figure 3C).

To verify the preventive or therapeutic effect of polyI:C in AP, we tested multiple time windows of polyI:C injection. PolyI:C administration 1 h prior to the induction of AP by caerulein effectively inhibited pancreatic injury (Supplementary Figures 4A,B), and also suppressed serum amylase and lipase induction by caerulein (Supplementary Figure 4C). However, polyI:C administration after induction of AP by caerulein in WT mice did not show a significant protective effect (data not shown).

Thus, we have found that the TLR3 ligand polyI:C effectively and safely prevents caerulein-induced AP and L-Arginine-induced AP in WT mice.

### IFN-β/IFNAR Signaling Is Required for the Preventive Effect of PolyI:C on AP

**Figure 2:**
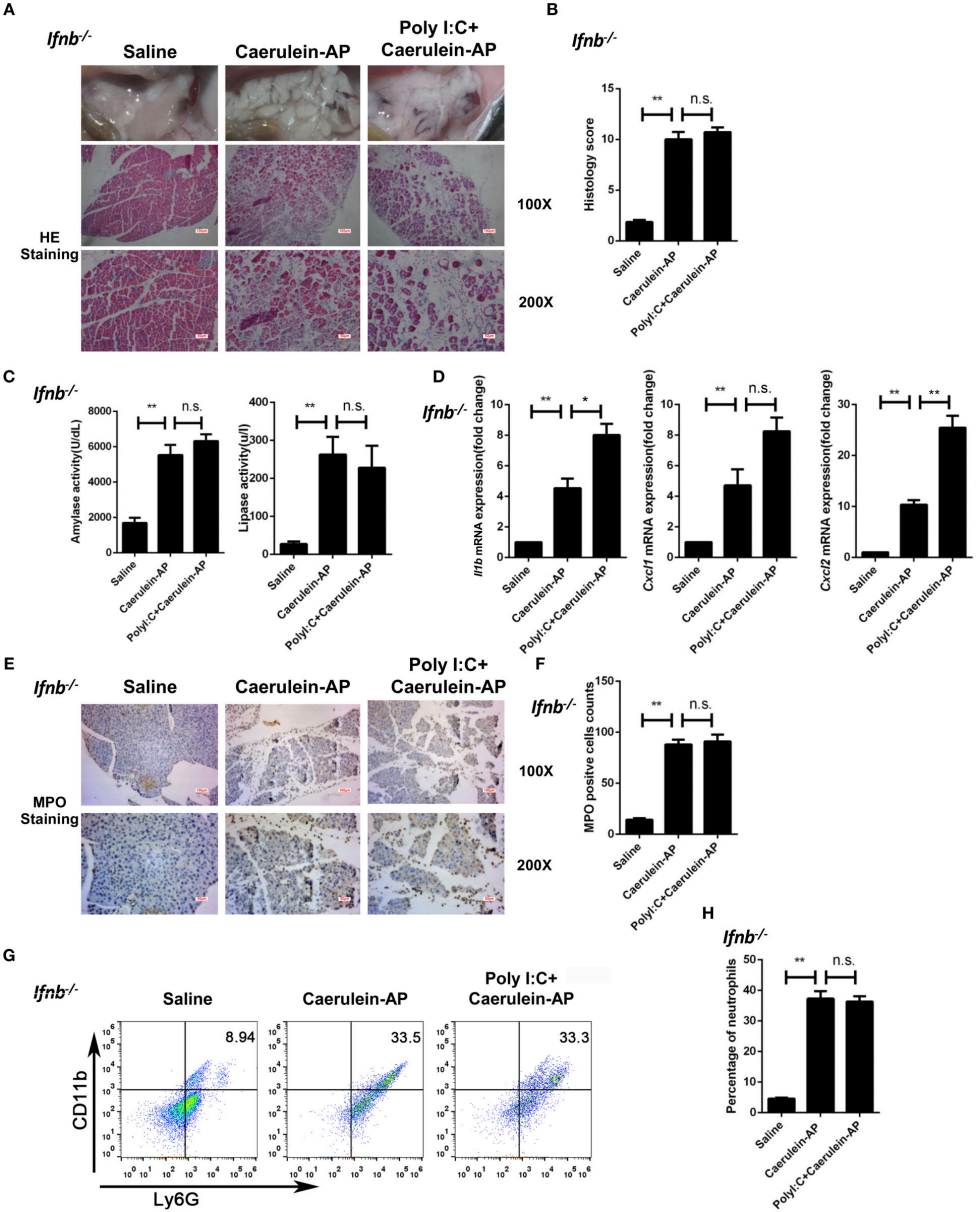
Knockout of Ifnb abolishes the protective effects of polyI:C on the caerulein-induced AP mouse model. **(A)** Histopathological examination of the effect of polyI:C on *Ifnb*^−/−^ caerulein-induced experimental AP mouse models. Top panel: gross observation; middle panel: H&E staining, 100X magnification; bottom panel: H&E staining, 200X magnification. **(B)** Histology scores of pancreatitis were evaluated and compared after observing five separate fields. **(C)** Activities of the serum amylase (left) and lipase (right) from Saline, Caerulein-AP, and PolyI:C+Caerulein-AP *Ifnb*^−/−^ mice were compared via enzymatic methods. **(D)** mRNA expression levels of *Il1b, Cxcl1*, and *Cxcl2* genes in the pancreatic tissue from Saline, Caerulein-AP, and PolyI:C+Caerulein-AP *Ifnb*^−/−^ mice were detected by RT-qPCR and normalized to *Rpl32*. **(E)** Neutrophil infiltrations in the pancreases from *Ifnb*^−/−^ caerulein-induced experimental AP mouse models were measured and compared by MPO staining. Top panel: 100X magnification; bottom panel: 200X magnification. **(F)** MPO^+^ cells were counted and compared after observing five separate fields. **(G)** Pancreases neutrophils from the Saline, Caerulein-AP, and PolyI:C+Caerulein-AP *Ifnb*^−/−^ mice were analyzed by flow cytometry. CD11b^+^Ly6G^+^ cells were considered as neutrophils. **(H)** The percentage of neutrophils from indicated groups were calculated and compared. Data of **(A,E,G)** are representative of three independent experiments. Data of **(B,F)** are shown as mean ± SD (*n* = 5) from one representative experiment. Data of **(C,D,H)** are shown as mean ± SEM from at least three independent experiments. ^*^p < 0.05, ^**^p < 0.01, n.s., not significant, one-way ANOVA test.

To confirm whether polyI:C-triggered type I IFN production mediated the protective effect of polyI:C in AP mice, we pretreated *Ifnb*^−/−^ mice with polyI:C in the caerulein-induced AP mouse model. PolyI:C did not alleviate the AP symptoms such as pancreas edema, inflammatory cell infiltration, and tissue necrosis in the *Ifnb*^−/−^ mice (Figures 2A,B). Consistently, polyI:C did not inhibit the elevation of serum amylase and lipase in the *Ifnb*^−/−^ experimental AP mice (Figure 2C). In addition, we found even higher inflammatory cytokine and chemokine gene expression (such as IL-1β and CXCL2) in the polyI:C-pretreated *Ifnb*^−/−^ AP mice, whereas polyI:C inhibited those inflammatory genes significantly in the WT AP mice (Figures 1E, 2D). The number of infiltrated neutrophils in *Ifnb*^−/−^ AP mice was indistinguishable between the saline and polyI:C pretreated groups (Figures 2E–H). These results indicate that polyI:C-triggered IFN-β production potentially meditates the preventive effect of polyI:C on AP.

**Figure 3:**
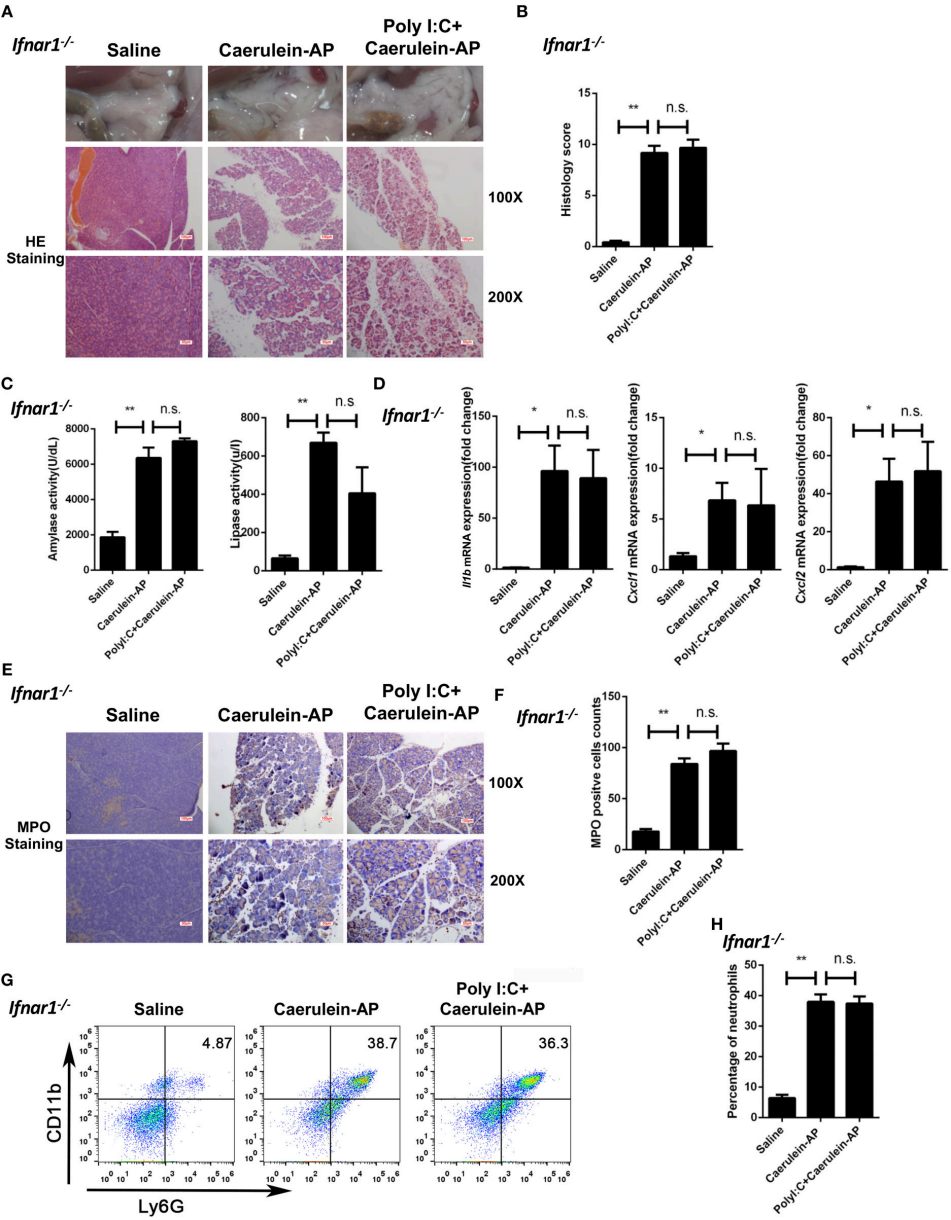
Knockout of Ifnar1 also abolishes the protective effects of polyI:C on the caerulein-induced AP mouse model. **(A)** Histopathological examination of the effect of polyI:C on *Ifnar1*^−/−^ caerulein-induced experimental AP mouse models. Top panel: gross observation; middle panel: H&E staining, 100X magnification; bottom panel: H&E staining, 200X magnification. **(B)** Histology scores of pancreatitis were evaluated and compared after observing five separate fields. **(C)** Activities of the serum amylase (left) and lipase (right) from Saline, Caerulein-AP, and PolyI:C+Caerulein-AP *Ifnar1*^−/−^ mice were compared via enzymatic methods. **(D)** mRNA expression levels of *Il1b, Cxcl1*, and *Cxcl2* genes in the pancreatic tissue from Saline, Caerulein-AP, and PolyI:C+Caerulein-AP *Ifnar1*^−/−^ mice were detected by RT-qPCR and normalized to *Rpl32*. **(E)** Neutrophil infiltrations in the pancreases from *Ifnar1*^−/−^ caerulein-induced experimental AP mouse models were measured and compared by MPO staining. Top panel: 100X magnification; bottom panel: 200X magnification. **(F)** MPO^+^ cells were counted and compared after observing five separate fields. **(G)** Pancreases neutrophils from the Saline, Caerulein-AP, and PolyI:C+Caerulein-AP *Ifnar1*^−/−^ mice were analyzed by flow cytometry. CD11b^+^Ly6G^+^ cells were considered as neutrophils. **(H)** The percentage of neutrophils from indicated groups was calculated and compared. Data of **(A,E,G)** are representative of three independent experiments. Data of **(B,F)** are shown as mean ± SD (*n* = 5) from one representative experiment. Data of **(C,D,H)** are shown as mean ± SEM from at least three independent experiments. ^*^p < 0.05, ^**^p < 0.01, n.s., not significant, one-way ANOVA test.

Next, we used the *Ifnar1*^−/−^ mice to confirm the above conclusion. The protective function of polyI:C was not seen in *Ifnar1*^−/−^ AP mice. Similar to the phenotypes observed in the *Ifnb*^−/−^ AP mice, there was no significant changes in pancreas histopathological results (Figures 3A,B), serum amylase and lipase activity levels (Figure 3C), pancreas inflammatory genes (Figure 3D), and neutrophil infiltration (Figures 3E–H) between the saline and polyI:C pretreated groups. In summary, these experiments suggest that IFN-β/IFNAR signaling is necessary for the preventive effect of polyI:C on caerulein-induced AP.

### PolyI:C Inhibits Oxidative Stress in AP in an IFN-β/IFNAR-Dependent Manner

**Figure 4:**
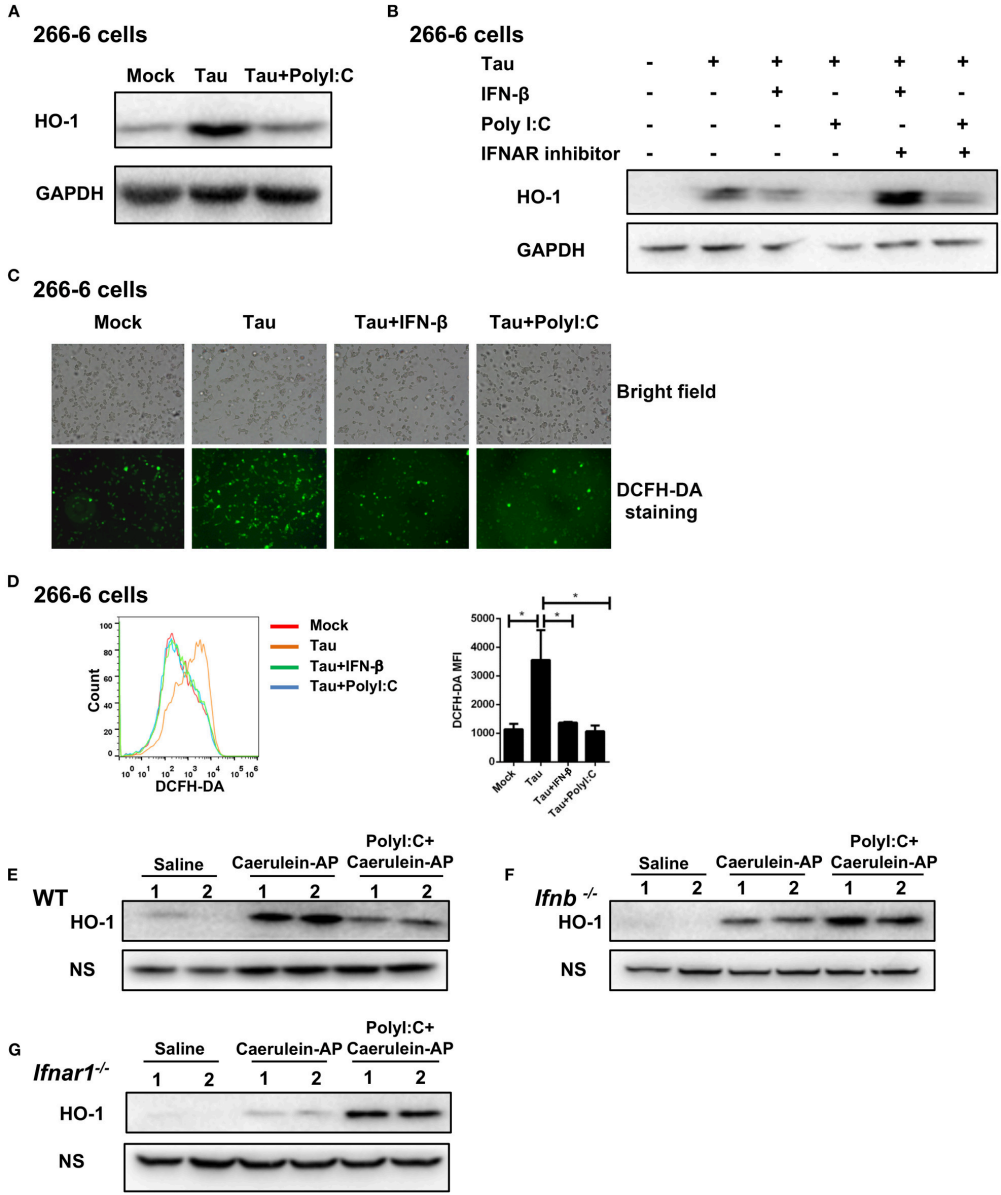
PolyI:C inhibits ROS production in AP via the IFN-β/IFNAR-dependent signaling pathway. **(A)** 266-6 cells were stimulated with 0.5 mM taurocholate in the absence or presence of polyI:C (1 μg/ml), 24 h later, HO-1 protein levels in the cells were measured by western blotting; GAPDH was used as a loading control. **(B)** 266-6 cells were stimulated with 0.5 mM taurocholate in the absence or presence of IFN-β (200 U/ml), polyI:C (1 μg/ml), or IFNAR inhibitor (1 μM). Twenty-four hours later, HO-1 protein levels in the cells were measured by western blotting; GAPDH was used as a loading control. **(C,D)** 266-6 cells were stimulated with 0.5 mM taurocholate in the absence or presence of polyI:C (1 μg/ml) or IFN-β (200 U/ml), intracellular ROS was stained by DCFH-DA, and detected by fluorescence microscopy **(C)** and flow cytometry **(D)**. Median fluorescence intensity (MFI) of stained DCFH-DA was calculated (**D**, right panel). **(E–G)** HO-1 protein expression levels in the pancreases from Saline, Caerulein-AP, and PolyI:C+Caerulein-A WT **(E)**, *Ifnb*^−/−^ **(F)**, and *Ifnar1*^−/−^ mice **(G)** were measured by western blotting; a non-specific (NS) band was shown as a loading control. Data of **(A–C,E–G)** are representative of three independent experiments. Data of **(D)**, right panel are shown as mean ± SEM (*n* = 3). ^*^p < 0.05, one-way ANOVA test.

The accumulation of ROS drives persistent tissue damage, resulting in the death of acinar cells, edema formation, and infiltration of inflammatory cells into the pancreas (25). Therefore, we tested whether polyI:C could inhibit the generation of ROS in oxidative injury-induced pancreatitis. As shown in Figure 4A, polyI:C treatment significantly suppressed the induction of anti-oxidant protein HO-1 by taurocholate in the 266-6 cells, however it did not suppress other anti-oxidant proteins such as KEAP1 and NQO1 (Supplementary Figure 5). Further experiments confirmed the role of the IFN-β/IFNAR signaling pathway in modulating ROS generation. PolyI:C or IFN-β treatment downregulated the induction of HO-1 protein by taurocholate, and blocking IFN-β and IFNAR interaction using an IFNAR inhibitor reversed the effect of polyI:C and IFN-β on HO-1 induction (Figure 4B).

HO-1 is not only an anti-oxidant protein for reducing ROS but can also be used as an indicator for measuring the severity of damage and inflammation in AP. Downregulation of HO-1 by polyI:C suggests the inhibition of oxidative stress. As we expected, polyI:C or IFN-β treatment significantly reduced the ROS level in taurocholate-stimulated 266-6 cells according to the fluorescence microscopy and flow cytometry results (Figures 4C,D).

Furthermore, we confirmed that polyI:C inhibited the generation of ROS in the pancreases of AP mice via the IFN-β/IFNAR signaling pathway. Homogenates of pancreases resected from experimental AP models with or without polyI:C in WT, *Ifnb*^−/−^, and *Ifnar1*^−/−^ mice were analyzed by immunoblotting for HO-1 protein levels. PolyI:C pretreatment suppressed HO-1 induction in the WT AP mice (Figure 4E). However, even higher HO-1 protein levels were detected in the polyI:C-pretreated *Ifnb*^−/−^ and *Ifnar*^−/−^ AP mice (Figures 4F,G). These data suggest that IFN-β and IFNAR downstream signaling inhibits oxidative stress during AP progression, which could be indicated by higher HO-1 production. Our results suggest that polyI:C inhibits ROS generation independently of HO-1. Without IFN-β/IFNAR signaling, polyI:C cannot facilitate the clearance of ROS, and thus, the pancreas requires more HO-1 anti-oxidant protein. TLR4 was reported to be suppressed by the TLR3 ligand polyI:C and thus, alleviates liver injury induced by LPS and D-GalN (14). However, in the caerulein-induced AP model, polyI:C protected the pancreas of AP mice from injury and inhibited the induction of amylase and lipase, although it was not as effective as in WT mice (Supplementary Figure 6).

By analyzing ROS levels and the amount of the oxidative stress indicator protein *in vitro* and *in vivo*, we have confirmed that polyI:C inhibits oxidative stress during AP progression in an IFN-β/IFNAR-dependent manner.

### PolyI:C Inhibits Multiple Genes Which Positively Correlated With AP Progression

**Figure 5:**
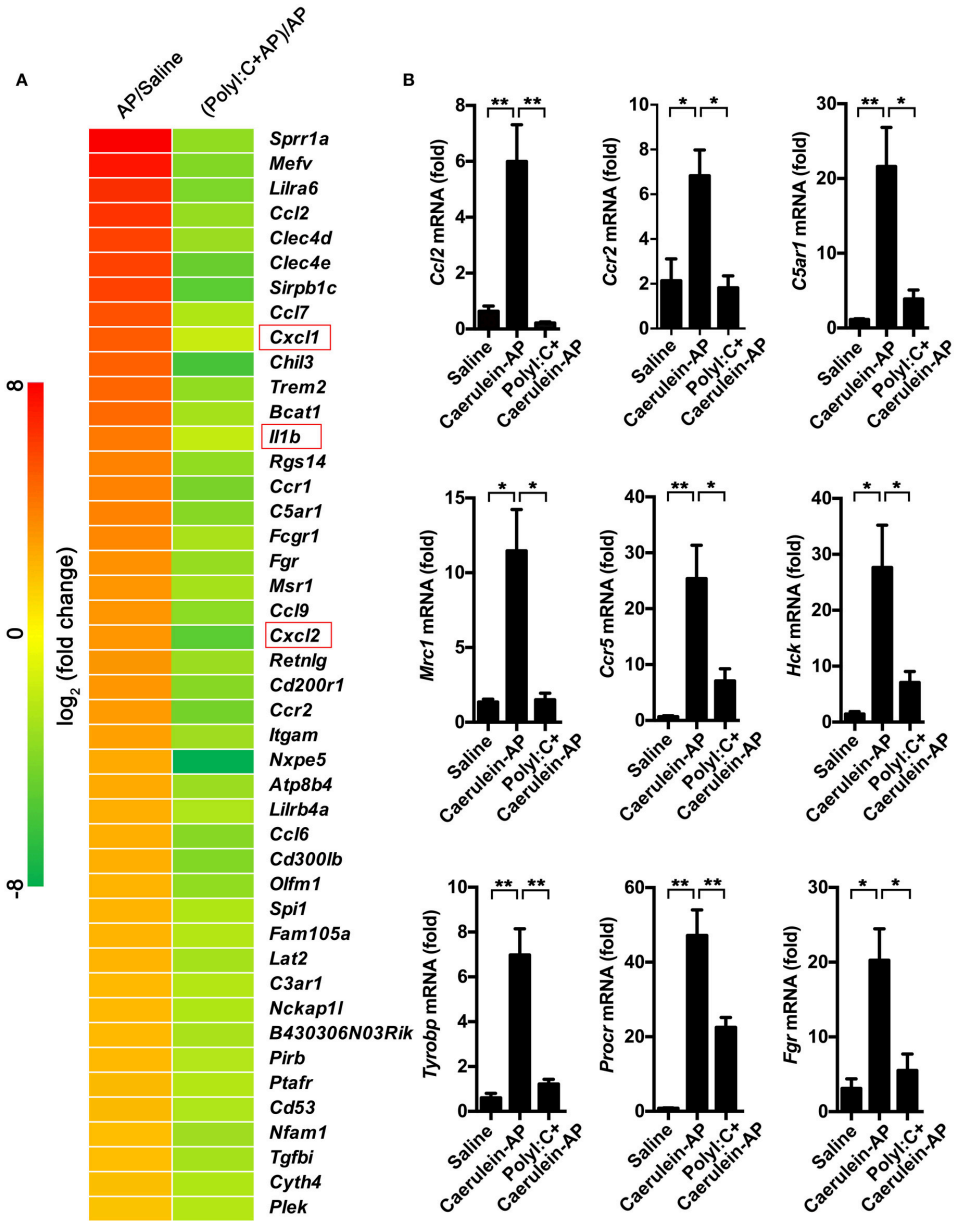
PolyI:C suppresses multiple genes related to the progression of AP. **(A)** Gene expression profiles of the pancreases from Saline, Caerulein-AP, and PolyI:C+Caerulein-AP WT mice were detected by RNA-Seq. Top polyI:C-suppressed and caerulein-inducible DEGs (differentially expressed genes) are shown in the heatmap. **(B)** Samples are treated as described in **(A)**, and mRNA expression level of the genes related to the progression of AP such as *Ccl2, Ccr2, C5ar1, Mrc1, Ccr5, Hck, Tyrobp, Procr*, and *Fgr* were verified by qPCR. Data of **(A)** are representative of three independent experiments. Data of **(B)** are shown as mean ± SEM (*n* = 3). ^*^p < 0.05, ^**^p < 0.01, one-way ANOVA test.

**Figure 6:**
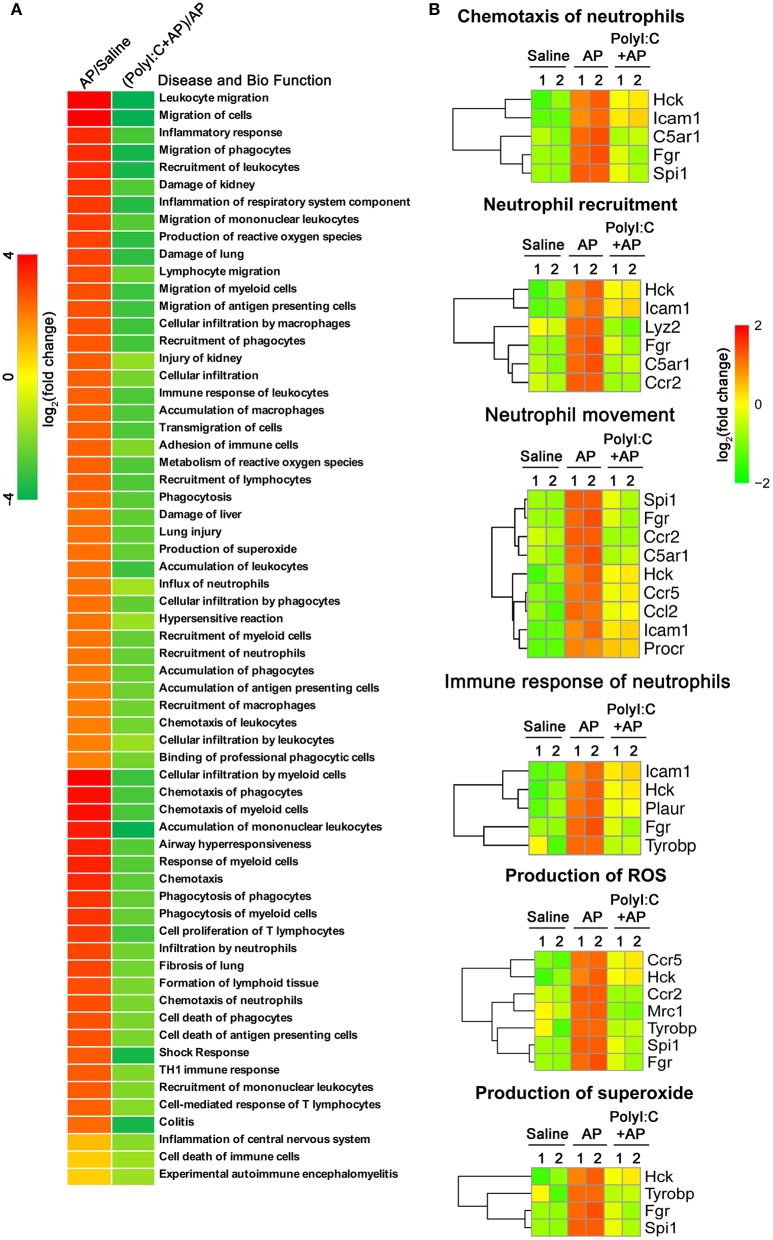
PolyI:C suppresses ROS production and neutrophil functions. **(A)** Gene expression profile of the pancreases from Saline, Caerulein-AP, and PolyI:C+Caerulein-AP WT mice was detected by RNA-Seq. DEGs were analyzed by the IPA and GO, and clustered into the pathways related to the disease and bio functions. **(B)** Genes related to the six pathways on ROS production and neutrophil functions are shown.

To explore the mechanism of action of polyI:C in preventing AP, we performed RNA-sequencing and analyzed the pancreas' transcriptomes from the saline-pretreated, AP, and polyI:C-pretreated AP mice. We focused on the genes that were significantly induced during AP and suppressed by polyI:C pretreatment. Among them were the inflammatory cytokine gene *Il1*β, the inflammatory chemokine genes *Cxcl1* and *Cxcl2*, and other genes, such as *Ccl2, Ccr2, C5a1, Mrc1, Ccr5, Hck, Tyrobp, Procr*, and *Fgr*, related to neutrophil recruitment, neutrophil movement, the immune response of neutrophils, and ROS production (Figure 5A). We verified some of the genes we were interested in and further analyzed all the differently expressed genes using IPA software (Figures 5B, 6). Key pathways and related genes that facilitated AP progression and were suppressed by polyI:C are shown in heatmaps (Figure 6).

**Figure 7:**
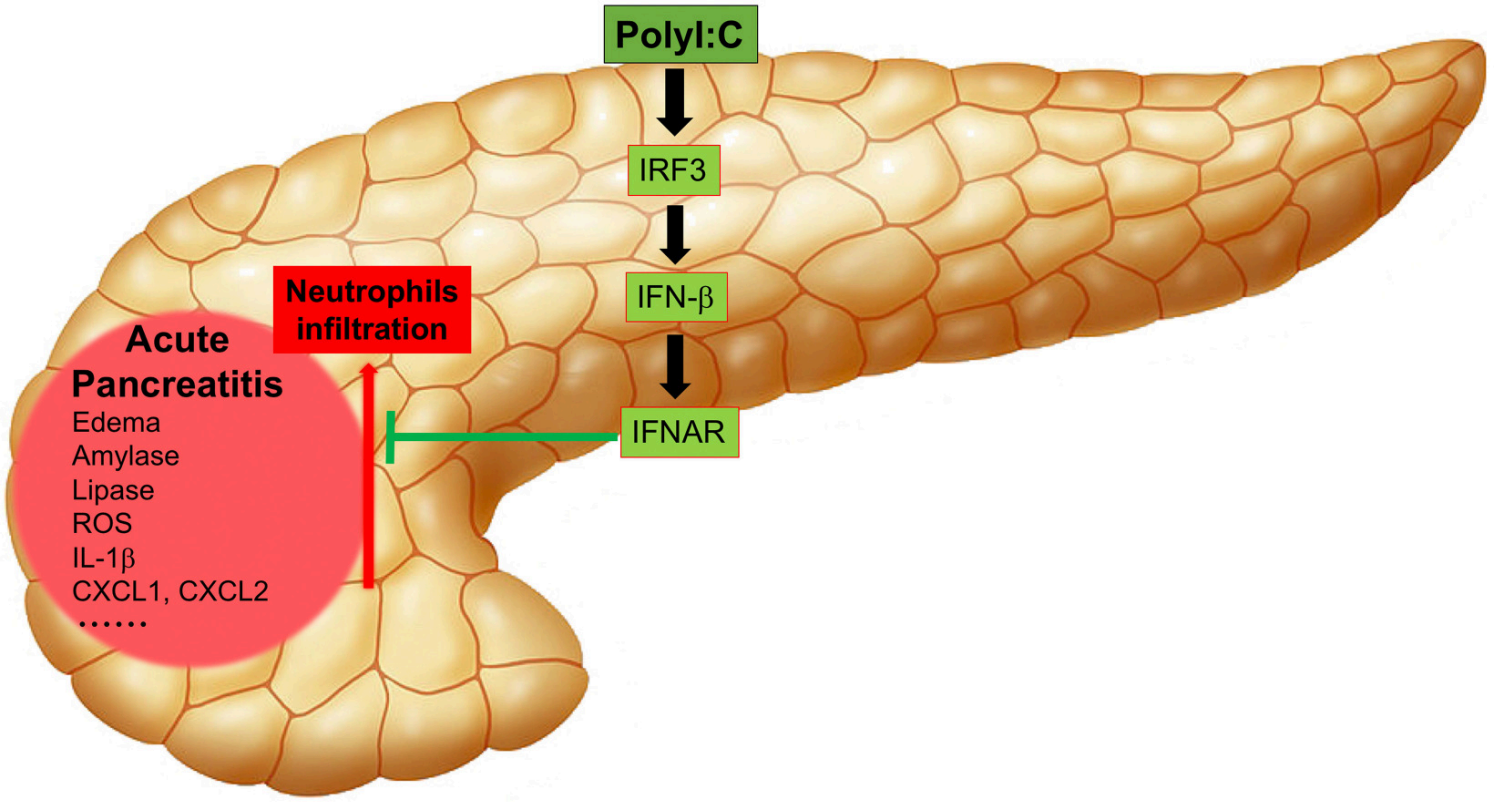
Working model of polyI:C-mediated protection on pancreases from inflammatory injuries. PolyI:C triggers the IFN-β production by activating the master transcription factor IRF3, and IFN-β inhibits neutrophil infiltration, and thus alleviates the AP symptoms such as edema, release of amylase and lipase, production of ROS, expression of the inflammatory genes by activating the downstream IFNAR signaling.

In summary, our study has shown that polyI:C triggers IFN-β production by activating the master transcription factor IRF3, and IFN-β inhibits neutrophil infiltration, thus, alleviating AP symptoms such as edema, release of amylase and lipase, generation of ROS, and the induction of inflammatory genes by activating downstream IFNAR signaling (Figure 7).

## Discussion

It has been well-established that type I IFN has a protective effect against viral infections by activating IFNAR downstream signaling and inducing IFN-stimulated genes (ISGs) (26). However, it is debated whether type I IFN plays beneficial or detrimental roles in inflammatory diseases (22). Unlike other TLRs such as TLR2, TLR4, and TLR9 usually promote inflammation (24), and TLR3 seems to act as the “peacemaker” in the maintenance of a healthy internal environment. It has been shown that TLR3 or TLR7 sense viral infection in the gut and trigger the production of IFN-β that dampens DSS-induced experimental colitis (27). Here, we have described an anti-inflammatory role of TLR3 in AP. We have found that pretreatment with TLR3 ligand polyI:C protects the caerulein-induced AP mice in an IFN-β/IFNAR-dependent manner.

Compared to the recombinant type I IFN, viral dsRNA mimic polyI:C is much more stable, easier to deliver, and has a lower cost. The use of TLR3 agonists as immunotherapeutic agents has been employed in cancer therapy to induce tumor cell apoptosis in type I IFN-dependent and independent pathways (28, 29). Pretreatment of polyI:C is very effective in preventing caerulein-induced AP and L-arginine-induced AP. In addition, polyI:C is safe for use in mice pancreases according to the non-pathogenic saline-injected mouse model. Given the efficacy and safety of polyI:C in protecting AP mice, it has shown the great potential of the application of TLR3 agonists to prevent AP.

In the AP models we used in this study, IFN-β was found to be responsible for most of the preventive effects of polyI:C on AP, whereas IFN-α seems to play a minor role in this process. Type I IFN is secreted from macrophages during AP progression, and IFN-β is predominantly secreted from macrophages, endothelial cells, and epithelial cells (30). Large amounts of IFN-α protein is produced by plasmacytoid dendritic cells under certain conditions (26). IFN-β is primarily produced during AP and induces the secondary wave of IFN-α via the well-established type I IFN positive feedback regulation loop (16–18). Most IFN-α genes are ISGs (31). Both IFN-β and IFN-α activate downstream signaling via the same receptor, IFNAR. However, the affinities of IFN-β and IFN-α for the receptor are different. IFN-β and IFN-α also induce the expression of different genes, which are expressed in macrophages stimulated with the same concentration of IFN-β and IFN-α (32, 33). In this study, we identified that IFN-β-induced genes play key roles in preventing AP.

Between 8 h and at least 1-h pretreatment of polyI:C before infection with caerulein or L-arginine is required for effective prevention of AP in the experimental mouse models. PolyI:C did not exert a good therapeutic effect on AP mice if polyI:C is injected post AP induction, since it takes over 6 h for polyI:C to induce the production of type I IFN. However, the level of serum amylase in human AP patients usually reaches its peak at around 48 h and returns to normal levels over the next 5–7 days (34), which is a much wider time window to administer polyI:C to human AP patients. Therefore, it is possible to use TLR3 agonists such as polyI:C as novel AP therapeutic agents if we treat AP patients with polyI:C at the early stages of disease.

It has previously been reported that polyI:C treatment significantly decreases mortality and liver injury caused by injection of LPS in the presence of D-GalN (14). PolyI:C also shows therapeutic effect against cerebral ischemia/reperfusion injury through the downregulation of TLR4 signaling (35). However, our results show that polyI:C protects *Tlr4*^−/−^ AP mice from pancreatic injury and inhibits the induction of amylase and lipase, although it is most effective in WT mice. In addition to IFN-β and downstream IFNAR signaling, it is possible that other polyI:C-responsive genes such as TLR4 also partially mediate the preventive effect of polyI:C on AP. Our future studies will aim to identify the ISGs stimulated by polyI:C that prevent AP.

PolyI:C treatment inhibited the induction of the inflammatory cytokine IL-1β as well as the chemokine genes including CXCL1 and CXCL2 in pancreases from AP mice. The infiltration of neutrophils but not of macrophages to the injured pancreases of AP mice was suppressed. In addition, a lower production of ROS was detected in both the acinar cell line and the injured pancreases of AP mice pretreated with polyI:C. This is consistent with the reduced induction of anti-oxidant protein HO-1 during AP progression. HO-1 usually acts as a protective effector to clear the elevated ROS in inflammatory cells (36). Suppressed generation of ROS by polyI:C leads to a weak induction of HO-1, which suggests polyI:C-triggered type I IFN production protects acinar cells and mice pancreases in a HO-1-independent manner. Pretreatment with polyI:C enhanced the induction of HO-1 protein production in pancreases from *Ifnb*^−/−^ and *Ifnar1*^−/−^ AP mice. This is consistent with the results that polyI:C did not protect the *Ifnb*^−/−^ and *Ifnar1*^−/−^ AP mice. ROS drives cytochrome C release, leading to ATP-dependent caspase activation and apoptosis which occurs in many cell types (37). Thus, there is a need to identify strategies to clear accumulated ROS during oxidative stress in AP. For example, type III IFN member IFN-λ regulated the activation of AKT in a non-translational manner independent of the STAT pathway to diminish ROS production (10). We have outlined a novel HO-1 independent pathway to clear ROS using type I IFN during AP progression.

AP is characterized by the activation of exocrine zymogen granules, which contain digestive enzymes (38), the infiltration of macrophage and neutrophils (39), and necrosis or apoptosis of pancreatic cells (40). Despite damaged primary pancreatic acinar cells, neutrophils isolated from patients with pancreatitis produce ROS to a greater extent (8, 41). Crosstalk among damaged cells, neutrophils, and ROS appears to synergistically promote the process of pancreatitis. According to the transcriptomic profiles of polyI:C-induced genes, we found that these genes were involved in the recruitment of inflammatory cells, production of ROS, and regulation of the inflammatory response. PolyI:C attenuated the positive feedback regulatory loop between ROS and proinflammatory cytokines in AP.

Inflammation occurring in the pancreas triggers multiple processes by recruiting inflammatory neutrophils and increasing ROS production, which leads to a homeostatic imbalance and sequentially promotes increasingly severe inflammatory injury. Regarding the protective role of polyI:C in preventing AP, TLR3 agonists are promising therapeutic agents to safely and effectively prevent AP at the early stages of pancreatitis.

## Ethics Statement

All animal procedures were approved by the Institutional Animal Care and Use Committee of Suzhou Institute of Systems Medicine.

## Author Contributions

FM and MZ conceived the idea. FM and CH designed the experiments. CH, SC, TZ, DL, ZH, JH, YQ, and BC finished all the experiments and data analysis. FM and CH wrote the manuscript. All authors contributed to the interpretation of the experiments, critically reviewed the manuscript, and gave the final approval of the manuscript submission.

### Conflict of Interest Statement

The authors declare that the research was conducted in the absence of any commercial or financial relationships that could be construed as a potential conflict of interest.
