# Supplementary material for: TLR3 Ligand PolyI:C Prevents Acute Pancreatitis Through the Interferon-β/Interferon-α/β Receptor Signaling Pathway in a Caerulein-Induced Pancreatitis Mouse Model
Source: Front Immunol. 2019 May 3;10:980. doi: 10.3389/fimmu.2019.00980 (PMC6509240; doi:10.3389/fimmu.2019.00980)
Supplement: Supplementary file 1 [file Data_Sheet_1.docx]

Supplementary Material

**Supplementary Figures and Tables**

**
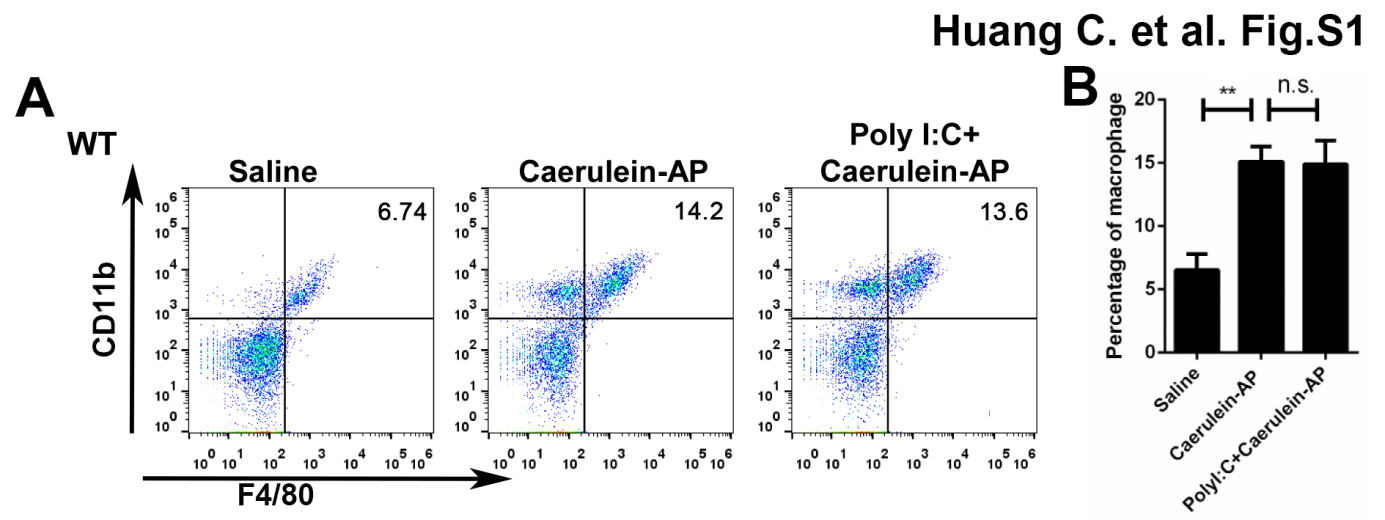
Supplementary Figures**

**Supplementary Figure 1.** **PolyI:C does not affect macrophage infiltration in the caerulein-induced AP mice. (A)** Flow cytometry detection of the infiltrated macrophages in the pancreas from Saline, Caerulein-AP, and PolyI:C+Caerulein-AP WT mice. CD11b^+^F4/80^+^ cells were considered as macrophages. Data are representative of three independent experiments. **(B)** The percentage of macrophages from indicated groups was calculated and compared. Data are shown as mean ± SEM from three independent experiments. ***p*<0.01, n.s.: not significant, one-way ANOVA test.


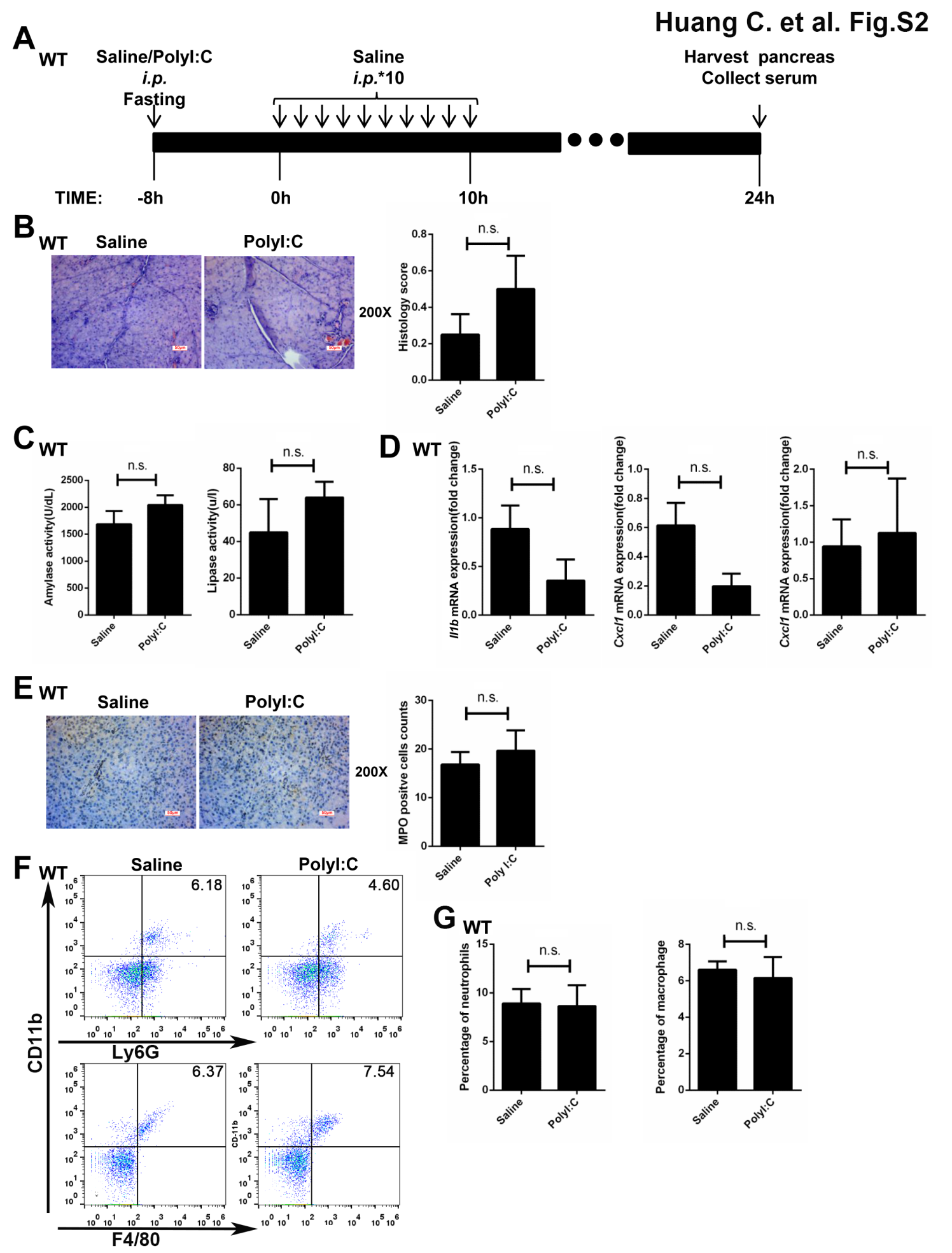


**Supplementary Figure 2.** **PolyI:C administration alone is safe. (A)** Mice were administrated intraperitoneally with saline or polyI:C (10 mg/kg) 8 h prior to 10 hourly injections of saline. **(B)** Histology of pancreatic sections from saline injected mice treated with saline or polyI:C; H&E staining, 200X magnification. Histology scores of pancreatitis were evaluated and compared after observing five separate fields, data are shown as mean ± SD (n=5) from one representative experiment. **(C)** Activities of the serum amylase (left) and lipase (right) from saline or polyI:C pretreated WT mice were compared via enzymatic methods. **(D)** mRNA expression levels of *Il1b*, *Cxcl1*, and *Cxcl2* genes in the pancreatic tissue from saline or polyI:C pretreated WT mice were detected by RT-qPCR and normalized to *Rpl32*. **(E)** Neutrophil infiltrations in the pancreases from saline or polyI:C pretreated WT mice were measured and compared by MPO staining. 200X magnification (left panel). MPO^+^ cells were counted and compared after observing five separate fields (right panel), data are shown as mean ± SD (n=5) from one representative experiment. **(F)** Neutrophils and macrophages in the pancreases from saline or polyI:C pretreated WT mice were analyzed by flow cytometry. CD11b^+^Ly6G^+^ cells were considered as neutrophils, and CD11b^+^F4/80^+^ cells were considered as macrophages. **(G)** The percentage of neutrophils (left panel) and macrophages (right panel) from indicated groups were calculated and compared. Data of **(B)**, **(E)**, and **(F)** are representative of three independent experiments. Data of **(C)**, **(D)**, and **(F)** are shown as mean ± SEM from at least three independent experiments. n.s.: not significant, unpaired student *t* test.


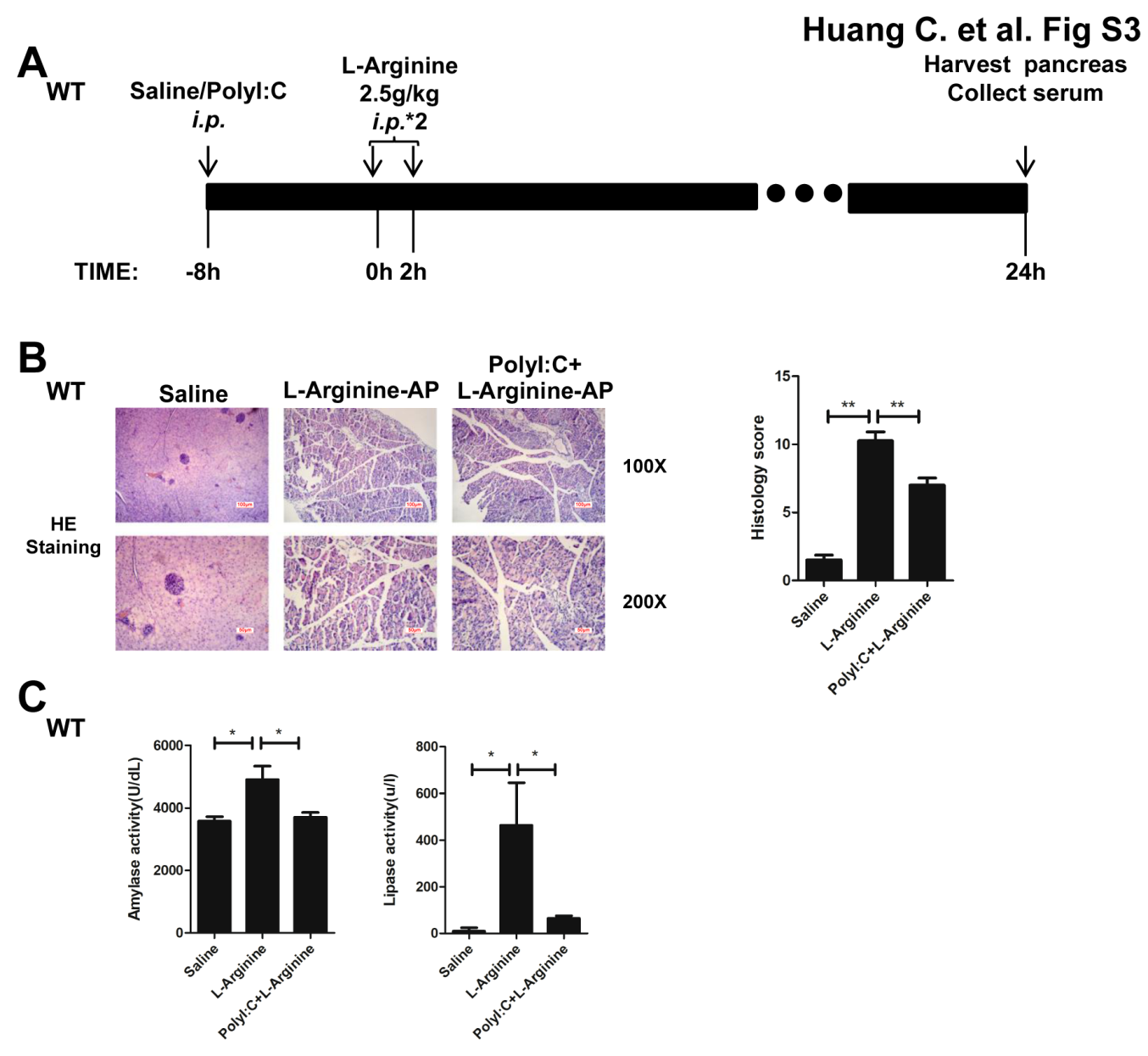


**Supplementary Figure 3.** **PolyI:C prevents L-arginine-induced AP in mice.** **(A)** Schematic diagram of the L-Arginine-induced experimental AP mouse model. Saline or polyI:C (10 mg/kg) was intraperitoneally administrated 8 h prior to the induction of AP. **(B)** Histopathological examination of the effect of polyI:C on L-Arginine-induced experimental AP WT mouse models by H&E staining. Top panel: 100X magnification; bottom panel: 200X magnification. **(C)** Histology scores of pancreatitis were evaluated and compared after observing five separate fields; data are shown as mean ± SD (n=5) from one representative experiment. **(D)** and **(E)** Activities of the serum amylase (**D**) and lipase (**E**) from Saline, L-Arginine-AP, and PolyI:C+L-arginine-AP WT mice were compared via enzymatic methods. Data of **(B)** are representative of three independent experiments. Data of **(D)** and **(E)** are shown as mean ± SEM (n≥3) from at least three independent experiments. **p*<0.05, ***p*<0.01, one-way ANOVA test.

**
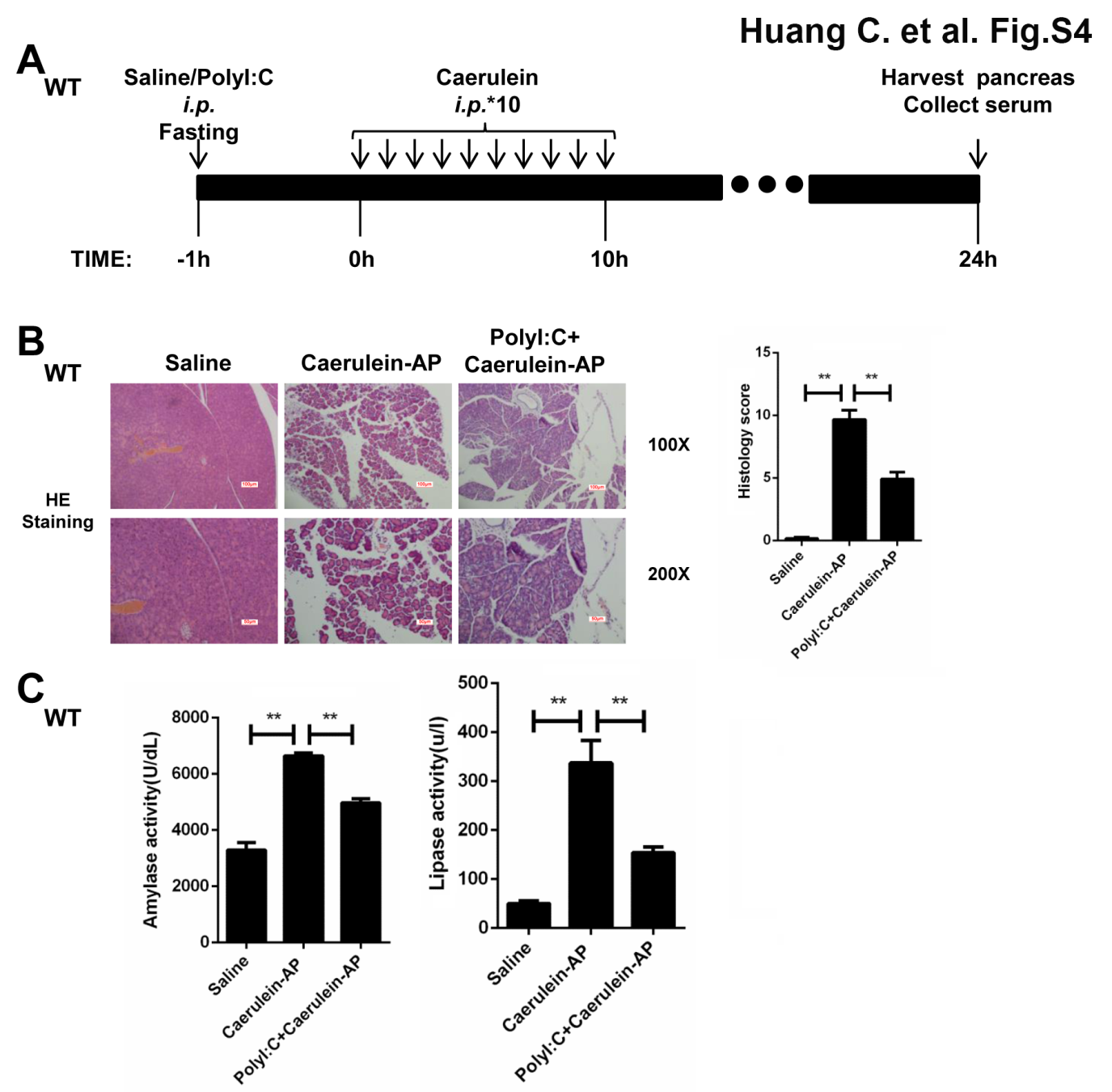
**

**Supplementary Figure 4.** **Short time pretreatment of polyI:C also prevents caerulein-induced AP in mice.** **(A)** Schematic diagram of the caerulein-induced experimental AP mouse model with 1 h pretreatment of polyI:C. **(B)** Histological examination of the effect of polyI:C (10 mg/kg, intraperitoneally administrated 1 h prior to the induction of AP on WT caerulein-induced experimental AP mouse models by H&E staining. Top panel: 100X magnification; bottom panel: 200X magnification. Histology scores of pancreatitis were evaluated and compared after observing five separate fields; data are shown as mean ± SD (n=5) from one representative experiment. **(C)** Activities of the serum amylase (left panel) and lipase (right panel) from Saline, Caerulein-AP, and PolyI:C+Caerulein-AP WT mice were measured via enzymatic methods. Data of **(B)** is representative of three independent experiments. Data of **(C)** are shown as mean ± SEM from at least three independent experiments. ***p*<0.01, one-way ANOVA test.


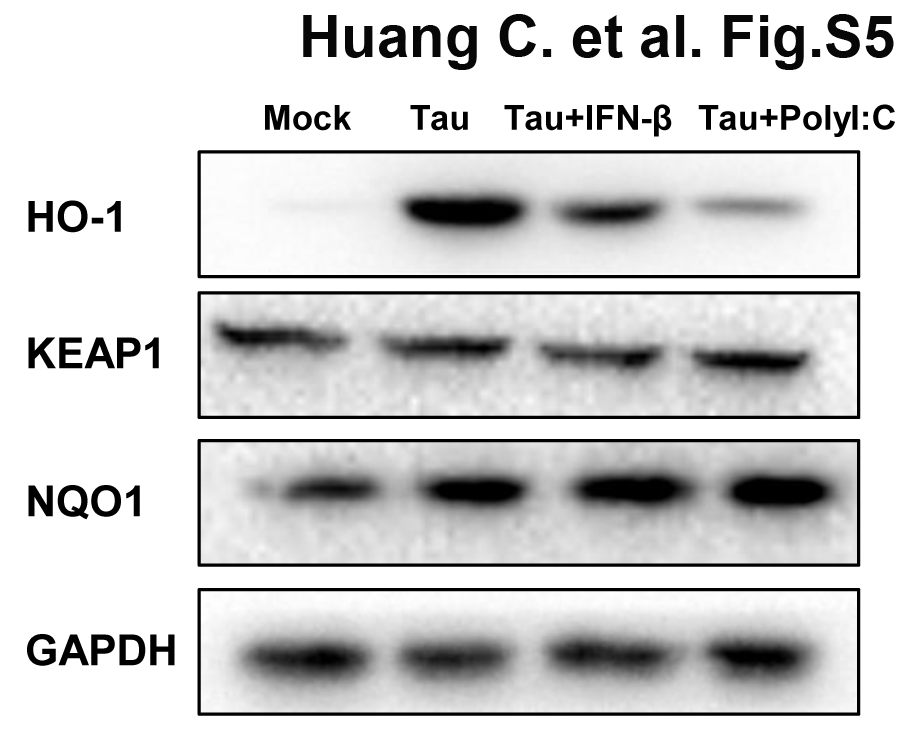


**Supplementary Figure 5. Anti-oxidant protein HO-1 is an indicator of the protective effect of polyI:C in the taurocholate-induced pancreatic acinar cell injury model.** 266-6 cells were stimulated with 0.5 mM taurocholate in the absence or presence of IFN-β (200 U/ml) or polyI:C (1 μg/ml), 24 h later, anti-oxidant protein levels **(**HO-1 KEAP1, NQO1) in the cells were measured by Western blotting, GAPDH was shown as a loading control. Data are representative of three independent experiments.


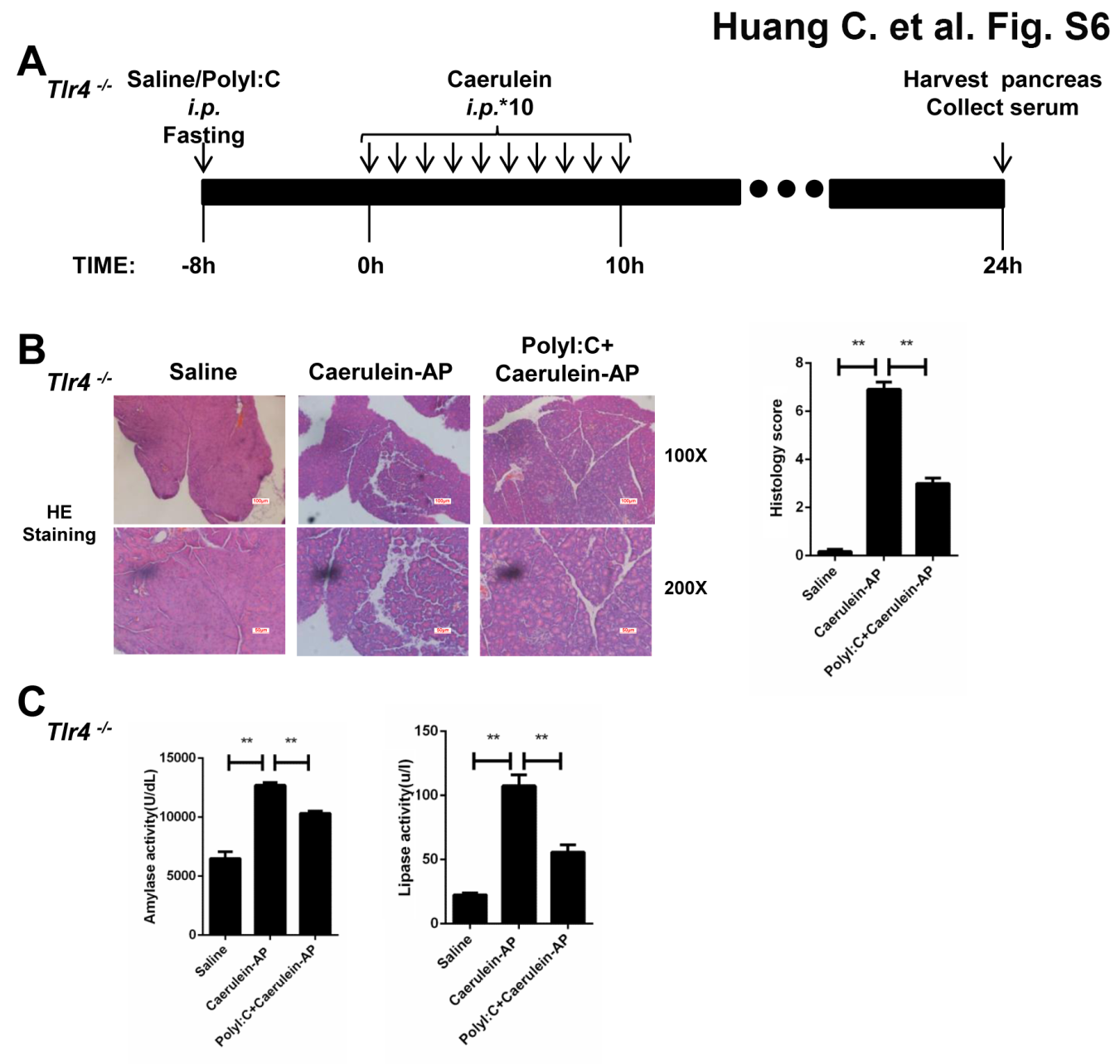


## Supplementary Figure 6. Knockout of *Tlr4* does not abolish the protective effects of polyI:C on the caerulein-induced AP mouse model. (A) Schematic diagram of the caerulein-induced experimental AP mouse model. Saline or polyI:C (10 mg/kg) was intraperitoneally administrated 8 h prior to the induction of AP in *Tlr4^-/-^* mice. (B) Histopathological examination of the effect of polyI:C on *Tlr4^-/-^* caerulein-induced experimental AP mouse models by H&E staining. Top panel: 100X magnification; bottom panel: H&E staining, 200X magnification. Histology scores of pancreatitis were evaluated and compared after observing five separate fields; data are shown as mean ± SD (n=5) from one representative experiment. (C) Activities of the serum amylase (left panel) and lipase (right panel) from Saline, Caerulein-AP, and PolyI:C+Caerulein-AP *Tlr4^-/-^* mice were compared via enzymatic methods. Data of (B) are representative of three independent experiments. Data of (C) are shown as mean ± SEM from at least three independent experiments. ***p*<0.01, one-way ANOVA test.

## Supplementary Tables

## Supplementary Table 1. Sequences of the primers used for quantitative real-time PCR.

| Gene | Primer list（5'-->3'） | |  |
| --- | --- | --- | --- |
| *L32* | Forward | GAAGTTCATCAGGCACCAGTC |  |
|  | Reverse | GAGCAATCTCAGCACAGTAAGA |  |
| *Il1b* | Forward | GACAGAACATAAGCCAACAA |  |
|  | Reverse | ACACAGGACAGGTATAGATTC |  |
| *Cxcl1* | Forward | AGACAGTGGCAGGGATTC |  |
|  | Reverse | TTCTTGAGTGTGGCTATGAC |  |
| *Cxcl2* | Forward | GCTCCTCAATGCTGTACT |  |
|  | Reverse | GAGTGGCTATGACTTCTGT |  |
| *Ccl2* | Forward | ATTCTGTGACCATCCCCTCAT |  |
|  | Reverse | TGTATGTGCCTCTGAACCCAC |  |
| *Ccr2* | Forward | ATCCACGGCATACTATCAACATC |  |
|  | Reverse | CAAGGCTCACCATCATCGTAG |  |
| *C5ar1* | Forward | ATGGACCCCATAGATAACAGCA |  |
|  | Reverse | GAGTAGATGATAAGGGCTGCAAC |  |
| *Mrc1* | Forward | CTCTGTTCAGCTATTGGACGC |  |
|  | Reverse | CGGAATTTCTGGGATTCAGCTTC |  |
| *Ccr5* | Forward | TTTTCAAGGGTCAGTTCCGAC |  |
|  | Reverse | GGAAGACCATCATGTTACCCAC |  |
| *Hck* | Forward | TCCTCCGAGATGGAAGCAAG |  |
|  | Reverse | ACAGTGCGACCACAATGGTAT |  |
| *Tyrobp* | Forward | GAGTGACACTTTCCCAAGATGC |  |
|  | Reverse | CCTTGACCTCGGGAGACCA |  |
| *Procr* | Forward | AATGCCTACAACCGGACTCG |  |
|  | Reverse | ACCAGTGATGTGTAAGAGCGA |  |
| *Fgr* | Forward | CGGCTGAAGAACGCTATTACC |  |
|  | Reverse | GGGCGACGAATATGGTCACTC |  |
|  | Reverse | TGTATGTGCCTCTGAACCCAC |  |

**Supplementary Table 2.** Histology scoring of pancreatitis

| **Edema** | **Acinar necrosis** |
| --- | --- |
| 0=absent | 0=absent |
| 0.5=focal expansion of interlobular septae | 0.5=focal occurrence of 1–4 necrotic cells/HPF |
| 1=diﬀuse expansion of interlobular septae | 1=diﬀuse occurrence of 1–4 necrotic cells/HPF |
| 1.5=same as1+focal expansion of interlobular septae | 1.5=same as 1+focal occurrence of 5–10necrotic cells/HPF |
| 2=same as 1+diﬀuse expansion of interlobular septae | 2=diﬀuse occurrence of 5–10 necrotic cells/HPF |
| 2.5=same as2+focal expansion of interacinar septae | 2.5=same as 2+focal occurrence of 11–16 necrotic cells/HPF |
| 3=same as 2+diﬀuse expansion of interacinar septae | 3 conﬂuent necrosis =diﬀuse occurrence of 11–16 necrotic cell~HPF or foci of |
| 3.5=same as 3+focal expansion of intercellular septae | 3.5=same as 3+focal occurrence of >16 necrotic cells/HPF |
| 4=same as3+diﬀuse expansion of intercellular septae | 4=>16 necrotic cells/HPF (extensive conﬂuent necrosis) |
| **Inﬂammation** | **Intrapancreatic hemorrhage** |
| 0=0–1 intralobular or perivascular leucocytes/HPF | 0=absent |
| 0.5=2–5 intralobular or perivascular leucocytes/HPF | 2=Focal occurrence in HPF |
| 1=6–10 intralobular or perivascular leucocytes/HPF | 4=Diﬀuse occurrence |
| 1.5=11–15 intralobular or perivascular leucocytes/HPF |  |
| 2=16–20 intralobular or perivascular leucocytes/HPF |  |
| 2.5=21–25 intralobular or perivascular leucocytes/HPF |  |
| 3=26–30 intralobular or perivascular leucocytes/HPF |  |
| 3.5=more than 30 leucocytes/HPF or focal microabscesses |  |
| 4=more than 35 leucocytes/HPF or conﬂuent microabscesses |  |
